# Nuclear CD24 promotes IL-6 secretion in fibroblasts

**DOI:** 10.1186/s12967-025-06477-4

**Published:** 2025-04-23

**Authors:** Yuqiao Sheng, Kexin Li, Shengli Ma, Chaoyang Zhang, Feng Li, Na Wei, Jingjing Xu, Rui Xue

**Affiliations:** 1 Medical Research Center, The First Affiliated Hospital of Zhengzhou University, Zhengzhou, 450052 Henan China; 2 Department of Emergency, The First Affiliated Hospital of Zhengzhou University, Zhengzhou, 450052 Henan China; 3 Department of Orthopedics, The First Affiliated Hospital of Zhengzhou University, Zhengzhou, 450052 Henan China; 4 Department of Pathology, The First Affiliated Hospital of Zhengzhou University, Zhengzhou, 450052 Henan China

Letter to the editor:

Cluster of differentiation (CD)24 is a glycosylphosphatidylinositol (GPI)-anchored glycoprotein. In cancer, CD24 is highly expressed, contributing to cell migration, invasion, and proliferation, and serves as a biomarker for poor prognosis [1]. Recent studies have identified new roles for CD24, showing that it protects tumor growth by interacting with the inhibitory receptor Siglec-10 on immune cells, sending a “don’t eat me” signal [2]. These findings highlight the surprising and prominent role of this small GPI-anchored cell surface molecule in biology.

Growing evidence indicates that CD24 is regulated by both intrinsic signaling pathways and extrinsic signals from the tumor microenvironment, which includes various stromal cells. Cancer-associated fibroblasts (CAFs), a major component of the tumor stroma, have been linked to CD24 expression on tumor cells, with studies showing a positive correlation between CD24 and α-SMA expression in CAFs. IL-6 secreted by CAFs can regulate CD24 expression on tumor cells by activating STAT3 phosphorylation and enhancing their stem cell properties [3]. Single-cell analysis has also identified CD24 + CAFs in tumor tissues, characterized by the expression of CD24 and the Wnt agonist gene RSPO3 [4]. These studies suggest a close connection between CD24 and fibroblasts, though the specific effects and mechanisms not yet fully understood.

To investigate CD24 expression in fibroblasts, we compared its levels in normal versus tumor-entrained fibroblasts (equivalent to CAFs). Our results showed that CD24 is significantly more expressed in tumor-entrained fibroblasts, not only on the cell membrane and in the cytoplasm but also in the nucleus (Fig. 1A, B). To confirm this, we performed subcellular fractionation on cell lysates and observed CD24 signals in the cytoplasm and membrane-bound fractions (M/Cyt) and nucleoplasmic fractions (Nuc) of 3T3 cells. Similarly, in fibroblasts trained with bladder cancer, CD24 expression in both parts was significantly increased (Fig. 1C). This is the first evidence of nuclear CD24 in fibroblasts, suggesting that it may shuttle between the plasma membrane and the nucleus, potentially regulating fibroblast growth and function at the genetic level.

**Fig. 1:**
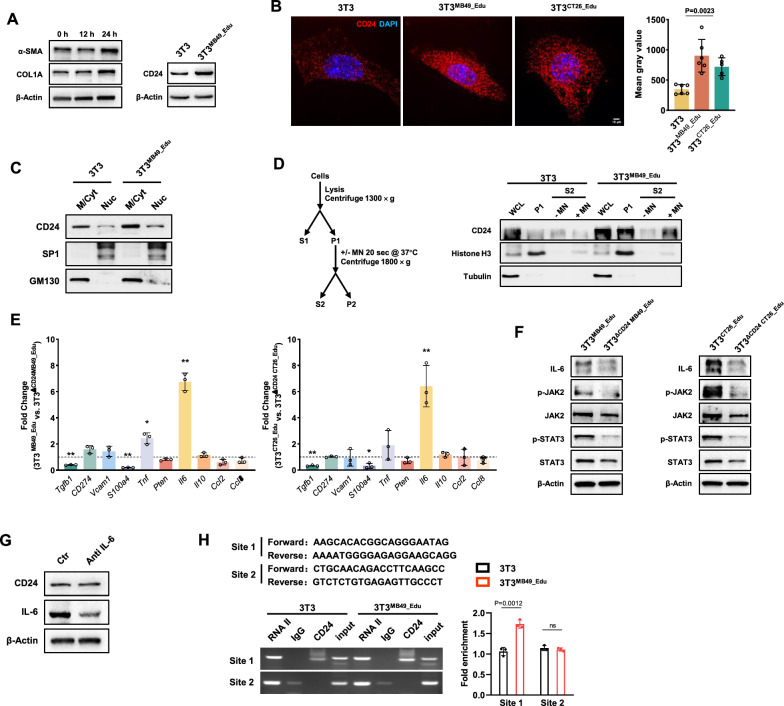
CD24 expression in tumor-associated fibroblasts and its role in IL-6 regulation. **A** Exposure of 3T3 cells to MB49 tumor-conditioned medium for 12 and 24 h increased α-SMA, a marker of tumor-associated fibroblasts, as well as CD24 protein levels in MB49-educated 3T3 cells. **B** Immunofluorescence analysis showed a significant increase in CD24 expression in both the plasma membrane and nucleus of 3T3 cells after being cultured with tumor cells (n = 5, p = 0.0023). **C** Cytoplasmic and nuclear proteins were extracted from 3T3 cells before and after tumor cell induction, followed by Western blot analysis. SP1 and GM130 were used as markers for the nucleus and cytoplasm, respectively, to observe changes in CD24 expression in these compartments. **D** A chromatin isolation strategy followed by DNA digestion with micrococcal nuclease (MN) were used. CD24 and histone H3 were both isolated from the chromatin preparation of 3T3 cells (P1) and subsequently released after DNA digestion (S2). **E** qPCR assessed the impact of CD24 knockout on cytokine and chemokine secretion by tumor-associated fibroblasts (n = 5, *p < 0.05, **p < 0.01). **F** Western blot analysis examined the IL-6/JAK2/STAT3 pathway activation in CAF cells before and after CD24 knockout. **G** After induction by tumor cells, 3T3 cells were treated with 25 ng/ml of the IL-6 neutralizing antibody Siltuximab for 24 h, and the expression levels of CD24 and IL-6 proteins were measured. **H** ChIP-qPCR assessed the enrichment of CD24 in the IL-6 promoter region in 3T3 cells before and after tumor induction (n = 3, p = 0.0012)

Next, we wanted to know whether CD24 can bind to chromatin, the main component of the cell nucleus. Our study revealed that both normal and tumor-educated 3T3 cells contained histone H3 and CD24 in the chromatin/insoluble protein fraction (P1). After DNA digestion with MN, histone H3 and CD24 were released (Fig. 1D), indicating that CD24, like histone H3, binds to chromatin, with a higher binding observed in tumor-educated 3T3 cells. However, due to the lack of known nuclear localization or export signals for CD24, it is currently not possible to explore the movement of CD24 in and out of the cell nucleus.

To understand CD24's role in fibroblasts, we analyzed the impact of CD24 knockout on chemokine and cytokine secretion in CAF cells (Fig. 1E). Our results showed that in fibroblasts after different tumor indoctrination, CD24 knockout significantly reduced the expression of IL-6 and inhibited the phosphorylation of its downstream molecules, JAK and STAT, after different tumor indoctrinations (Fig. 1F). Interestingly, the expression of CD24 was not affected by IL-6 antibodies (Fig. 1G), suggesting that CD24 may be upstream of IL-6.

To investigate further, we identified DNA fragments in the IL-6 promoter region linked to CD24 expression (Figure S1) and designed primers for these fragments to conduct ChIP-qPCR. As shown in Fig. 1H, CD24 protein successfully bound to the IL-6 promoter region in the nucleus of fibroblasts, confirming its role in regulating IL-6 gene expression. Additionally, the study found that after tumor cell education, the binding of CD24 to the IL-6 promoter region significantly increased, indicating that the tumor environment promotes this interaction in fibroblasts.

Although CD24 has traditionally been viewed as a cell surface glycoprotein, recent studies [5], including ours, have revealed its presence in other cellular locations with significant biological roles. We identified CD24 in fibroblasts and demonstrated its nuclear role in regulating IL-6 transcription. Notably, tumor-like conditions enhance CD24 expression and function in fibroblasts, suggesting broader roles than previously recognized. Our findings reinforce CD24's significance as a tumor driver and prognostic marker, particularly in CAFs, and highlight a new mechanism relevant to CD24-targeted therapies, emphasizing the importance of CD24 + fibroblasts in both research and clinical applications.

## Supplementary Information

Additional file 1. Figure S1. Chromatin analysis and IL-6 expression in CD24^high^ vs. CD24^low^ K562 cells.

## Data Availability

The datasets will be made available upon reasonable request.

## Declarations

### Animal studies

This study and included experimental procedures were approved by the institutional animal care and use committee of Zhengzhou University.
