# Supplementary figures and images for: Nuclear CD24 promotes IL-6 secretion in fibroblasts
Source: J Transl Med. 2025 Apr 23;23:464. doi: 10.1186/s12967-025-06477-4 (PMC12020148; doi:10.1186/s12967-025-06477-4)

Supplemental Figure 1

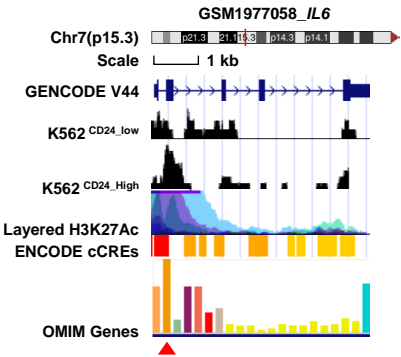

Supplement: Supplementary file 1 — Additional file 1. Figure S1. Chromatin analysis and IL-6 expression in CD24high vs. CD24low K562 cells. [file 12967_2025_6477_MOESM1_ESM.pdf]
